# Cryopreservation of Bone Marrow Mononuclear Cells Alters Their Viability and Subpopulation Composition but Not Their Treatment Effects in a Rodent Stroke Model

**DOI:** 10.1155/2016/5876836

**Published:** 2016-06-15

**Authors:** Bing Yang, Kaushik Parsha, Krystal Schaar, Nikunj Satani, Xiaopei Xi, Jaroslaw Aronowski, Sean I. Savitz

**Affiliations:** Stroke Program, Department of Neurology, McGovern Medical School, University of Texas Health Science Center, Houston, TX 77030, USA

## Abstract

The systemic administration of autologous bone marrow (BM) derived mononuclear cells (MNCs) is under investigation as a novel therapeutic modality for the treatment of ischemic stroke. Autologous applications raise the possibility that MNCs could potentially be stored as a banked source. There have been no studies that investigate the effects of cryopreservation of BM-MNCs on their functional abilities in stroke models. In the present study, C57BL/6 mice were subjected to middle cerebral artery occlusion (MCAo) for 60 minutes and then divided into two treatment groups: fresh MNCs versus cryopreserved MNCs. BM-MNCs were collected at 22 hours after MCAo and were stored in liquid nitrogen for 12 months in cryopreserved MNCs group. BM-MNCs cellular viability, composition, and phenotype of the various subpopulations of mice BM-MNCs were evaluated by flow cytometry, and the behavioral recovery of stroke animals was tested with freshly harvested MNCs versus cryopreserved MNCs by corner test and ladder rung test. We found that long-term cryopreservation negatively impacts the cellular viability of bone marrow MNCs. Cryopreservation also alters the cellular composition of various subpopulations within the MNCs. However, despite the changes observed in cryopreserved cells, both fresh and frozen MNCs have similar beneficial effect on behavioral and histological outcomes.

## 1. Introduction

The systemic administration of autologous bone marrow (BM) derived mononuclear cells (MNCs) is under investigation as a novel therapeutic modality for the treatment of ischemic stroke [1, 2]. Multiple preclinical studies have shown the benefits of neurofunctional recovery after stroke in experimental animal models [3–5] and several clinical trials have been undertaken to test their safety and preliminary efficacy in stroke patients [1, 2, 6, 7]. MNCs may reduce inflammation and amplify endogenous repair mechanisms after stroke [1]. The use of autologous cells from the bone marrow raises the possibility that MNCs could potentially be stored as a banked source for possible therapeutic use when the need arises. The prototypical example for such an application is autologous umbilical cord blood (UCB) derived cells [8, 9]. The process of cryopreservation, however, may alter the cellular composition and/or function and activity of certain types of cell therapies [10–12], which may affect their underlying efficacy. The effects of cryopreservation and thawing on bone marrow MNCs have not been thoroughly studied. It has been reported that cryopreserved human bone marrow MNCs do not alter the ability of osteoblastogenesis as a mesenchymal stromal cells source compared to fresh collected MNCs [13]. Xie et al. reported that cryopreserved MNCs maintain their bioactivity and benefits for promotion of bone repair in a rabbit model with steroid-associated osteonecrosis [14]. However, there have been no studies that have investigated the effects of cryopreservation of BM-MNCs on their functional abilities in stroke models. We therefore conducted this study to evaluate the effects of cryopreservation on cellular viability, composition, and phenotype of the various subpopulations of mice BM-MNCs and test the effect of cryopreservation on behavioral recovery of ischemic animals administered intravenously with fresh versus cryopreserved MNCs.

## 2. Methods

### 2.1. Animals

39 male C57 BL/6 mice (Harlan Laboratories, USA) between 8 and 10 weeks old were used in this study. All animals were double housed with free access to food and water. Subjects were maintained on a standard 12 : 12 h light/dark cycle. All outcome assessments and data analyses were completed blinded to treatment groups. All procedures were approved by the UT-Houston Health Science Center Animal Welfare Committee.

### 2.2. MCA Occlusion

Focal ischemia of 60 min duration in male C57BL/6 mice was induced by suture occlusion of the middle cerebral artery (MCAo) with Longa's method [15]. Animals were anesthetized with 2% isoflurane in a mixture of N_2_O/O_2_ (50%/50%). The common cerebral artery (CCA) is temporarily closed with a vessel clip. A 6-0 nylon monofilament with a heated blunt tip was introduced through the left external carotid artery and advanced to the beginning of the left middle cerebral artery (MCA). The rectal temperature was monitored and controlled at 36.5 ± 0.5°C using a feed-forward temperature controller. After incision closure, a dose of 0.5% bupivacaine was given to minimize animal's pain and discomfort during recovery period. Cerebral blood perfusion (CBF) was monitored with a laser Doppler flowmeter (LDF) placed over the MCA territory (1 mm posterior and 3 mm lateral to the bregma on the left parietal skull), and only mice with ≥75% CBF reduction compared to baseline were confirmed to have successful occlusion and included in this study.

### 2.3. Isolation of BM-MNCs

The C57BL/6 male mice (8–10 weeks old) were subjected to transient MCAo by suture occlusion for 60 mins. Twenty-two hours later, donor mice were sacrificed and bone marrow (BM) was obtained from femurs and tibias. BM was flushed out by cold phosphate-buffered saline (PBS) + 0.5% BSA, dispersed by pipetting, and BM-MNCs were isolated by density gradient centrifugation with Ficol-Paque Plus [16]. The isolated MNCs were either cryopreserved (frozen) or used fresh to administer IV to a recipient mice with stroke. The use of an inbred strain of mice helped simulate some of the conditions of an autologous application. Four donors were employed to generate enough cryopreserved MNCs and another 3 donors were employed for fresh MNCs. Donor mice were euthanized under 5% fatal isoflurane.

### 2.4. Cryopreservation

MNCs were isolated as described above and placed in the cryovial with 90% FBS + 10% DMSO at 10^7^ cells/mL. Then, the vials were put in −20°C freezer for one hour, followed by sitting in −80°C freezer for 24 hour, and eventually transferred to liquid nitrogen tank for long-term storage (12 months).

### 2.5. MNCs Treatment and Groups

C57 BL/6 male mice were subjected to transient MCAo by suture occlusion for 60 mins as described above. 22 hours later, mice were randomly divided into three treatment groups after the first neurofunctional test evaluation after stroke: (1) saline, (2) fresh MNCs, and (3) cryopreserved MNCs. Mice received 3 × 10^7^/kg MNCs (fresh or frozen) with 100 *μ*L saline or only 100 *μ*L saline IV via the tail vein at 24 hours after stroke. The cells were infused at a rate of 0.02 mL/min over 5 minutes with the help of an autoinjection pump. Mice received either fresh MNCs or cryopreserved MNCs. The dosage was determined from our previous dosage study in the rat model [17]. In cryopreserved MNCs treatment group, the frozen MNCs were thawed in 37°C water bath and washed in DMEM, followed by PBS, and finally suspended in saline prior to cell counting and treatment.

### 2.6. Cell Characterization

The BM-MNCs were tested for viability, cellular composition, and phenotypes by using flow cytometry before and after their cryopreservation period. Briefly, 1 × 10^6^ MNCs were suspended in 100 mL Hank's Balanced Salt Solution + 2% FBS and then were incubated with anti-mouse antibodies specific for CD3-FITC, CD8-PE, CD4-PE, CD45-APC, and CD220-PE (1 : 100; Biolegend); CD11b-FITC, CD133-FITC, and CD335-PE (1 : 100; BD Bioscience); CD34-PE (1 : 5; Santa Cruz Biotechnology). Cell viability was assessed by exclusion of 7-amino actinomycin (7-DAA). Stained cells were collected using Gallios Flow Cytometer (Beckman Coulter) and analyzed with Kaluza software (Beckman Coulter). In cryopreserved MNCs treatment group, the frozen MNCs were thawed in 37°C water bath for less than 1 min and washed in DMEM, followed by PBS prior to the assay. To mimic the clinical condition, cell characterization was carried out by selecting specific antibodies which are known to be the closest to human expression [18–22].

### 2.7. Behavioral Testing

To evaluate the behavioral outcomes of mice after stroke which systemically received fresh or cryopreserved MNCs, we used previously validated [23] corner test and the ladder rung test. Mice were pretested before MCAo and before cell administration after MCAo and then tested on days 7, 14, 21, and 28 after stroke. All tests were performed by an examiner blinded to treatment allocation.

#### 2.7.1. Corner Test

Mice were allowed to walk into a corner formed by two Plexiglas boards, each 20 cm × 30 cm and fused into a 30-degree angle. As the mouse neared the corner, the two boards delivered bilateral vibrissae stimulation, and the mouse would rear up and turn out of the corner. The direction of rearing and turning was recorded for 10 trials. Each turn must have been preceded by a rear for the trial to be recorded. Trials in which the mouse turned without rearing were not recorded. The percentage of ipsilesional turns was calculated using the following formula: ipsilesional turns/10.

#### 2.7.2. Ladder Rung Test

Mice were videotaped walking across a horizontal ladder. The ladder rung apparatus was composed of an elevated horizontal ladder (80 cm long and 12 cm in elevation). The rungs were 1 mm in diameter and spaced evenly apart, and the ladder had Plexiglas sides that were 15 cm tall. For each trial, the mouse was placed on one end of the ladder and videotaped while walking across. The percent error (total faults/total steps) on the impaired side was scored.

### 2.8. Lesion Size

Twenty-eight days after stroke, mice were anesthetized under 5% isoflurane and intracardially perfused with ice-cold PBS, followed by ice-cold 4% paraformaldehyde (PFA) in PBS, and decapitated. Brains were harvested, postfixed in 4% PFA in PBS for 24 hours, immersed in 20% sucrose for 2 days, and divided into 6 coronal sections (1.2 mm for each). Coronal 20 *μ*m frozen slices from each of the sections were cut and stained with cresyl violet. A total of 6 sections per brain were taken and brain loss was calculated using Image J. As we reported previously [24], brain loss of the ipsilateral chronic infarct was measured using the indirect method and expressed as a percentage of the contralateral hemisphere by a researcher blinded to treatment groups: brain loss (percentage) = (the volume of contralateral hemisphere − ipsilateral hemisphere)/the volume of contralateral hemisphere × 100%.

### 2.9. Statistical Analysis

All data are presented as means ± SD. Repeated-measures ANOVA and Bonferroni posttest were used for comparison across groups at different time points after stroke in the behavioral tests. For lesion size assay, a one-way ANOVA was performed with post hoc Tukey-Kramer test. For cell characterization, *t*-test was performed after Rank Sum Test. Statistical significance was set at *p* < 0.05 level. The statistical analysis was performed by Sigma Blot 11.0.

## 3. Results

### 3.1. Mortality

32 mice were successfully subjected to stroke and randomly assigned to three treatment groups: saline: *n* = 12; fresh MNCs: *n* = 10; cryopreserved MNCs: *n* = 10. In the first 3 days after stroke, 2 mice died in each group and there was no significant difference in mortality across groups. There was no further mortality from day 3 to day 28 after stroke in the experiment.

### 3.2. Effects of Cryopreservation

**Figure 1:**
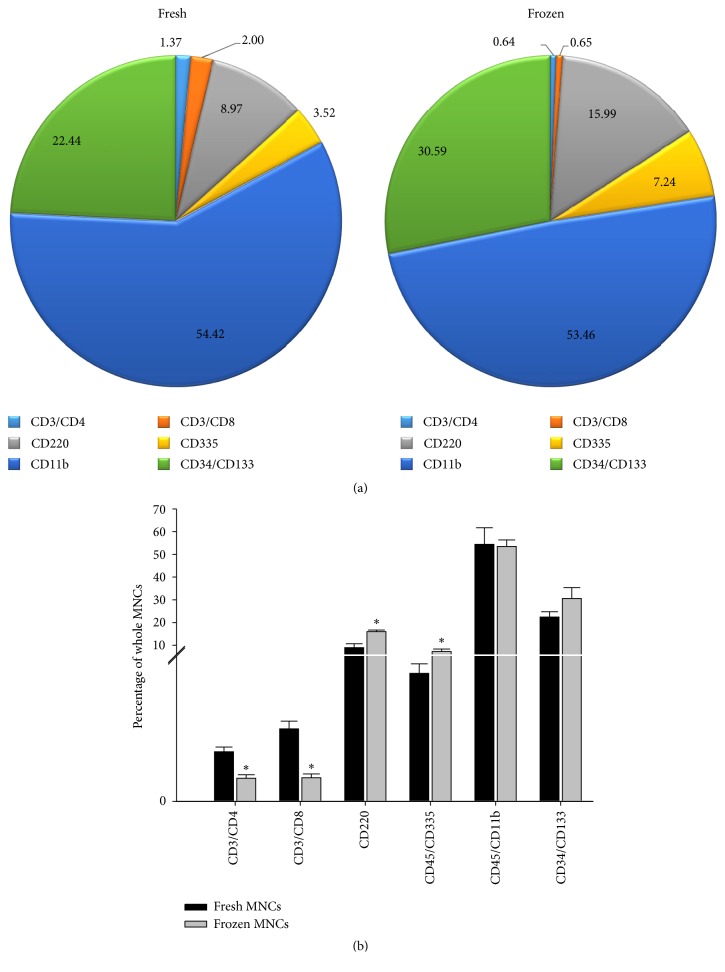
(a) Pie charts illustrating the mean percentage of certain cellular subpopulations within the whole MNCs. (b) Bar graphs exhibiting the significant changes in cellular subpopulation between fresh and cryopreserved MNCs group. Data are mean ± SD. *∗*: *p* < 0.05, fresh MNCs versus frozen MNCs. *n* = 4.

As we intended to mimic the possible clinical scenario of using cells from a cell bank, we tested the use of mouse BM-MNCs that were cryopreserved for 12 months. First, we tested whether cryopreservation and cell thawing would alter their viability. We found that the viability of MNCs was significantly decreased. Fresh MNCs had 95% ± 0.74 viability while cryopreserved MNCs had 78% ± 1.8 viability (*p* < 0.05). Then, we measured the changes in cellular composition and phenotypes within MNCs. Within MNCs, the proportion of both CD3+/CD4+ and CD3+/CD8+ (T cell marker) component was reduced significantly in the cryopreserved group, compared with fresh MNCs (*p* = 0.011, *p* = 0.010, resp.), but the CD220 (B cell marker) and CD45+/CD335+ cell population (Nature Killer cell marker) significantly increased (*p* = 0.037, *p* = 0.040, resp.). We did not observe significant differences in the proportion of CD45+/CD11b+ (monocytes marker) cell subpopulation between the two groups (*p* = 0.911). We further explored within CD45+/CD11b+ cellular populations with Ly6G and Ly6C. Compared to fresh MNCs, cryopreservation caused a tendency (*p* = 0.09) toward reduction in Ly6G+/Ly6C− granulocytic cells within CD45+/CD11b+ cells (11.6% ± 1.77% in frozen MNCs versus 21.67% ± 3.74% in fresh MNCs) (Figures 1(a) and 1(b)). Interestingly, cryopreservation also had no effect on the progenitor (CD34+/CD133+) subpopulations (*p* = 0.196).

### 3.3. Behavioral Outcome

**Figure 2:**
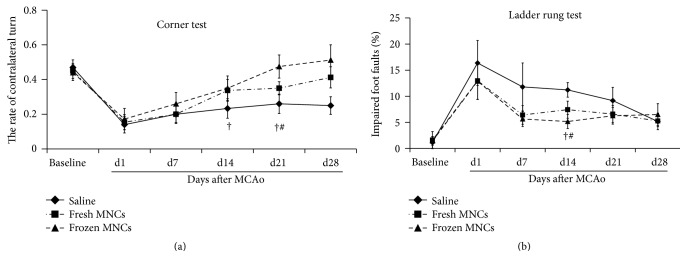
Line diagrams illustrating the neurofunctional improvement in mice treated with MNCs IV at 24 hours after stroke. Animals were assigned to 3 treatment groups: saline (*n* = 10), fresh MNCs (*n* = 8), or frozen MNCs (*n* = 8). All animals were evaluated on the corner test (a), where smaller value in *y*-axis meant more severe deficits and ladder rung tests (b), where larger value in axis meant more severe deficits, up to 28 days after stroke. Data are mean ± SD; †: *p* < 0.05, frozen MNCs versus saline; #: *p* < 0.05, fresh MNCs versus saline.

Since cryopreservation altered MNCs viability and composition, which might be associated with the beneficial or detrimental effects to stroke recovery by MNCs, we then examined differences in the effects on behavioral outcomes between two MNC groups. In the corner test, frozen as well as fresh MNC administration compared with saline treatment significantly improved neurological outcomes at 28 days after stroke. In the ladder rung test, the use of either fresh or frozen MNCs led to significant behavioral improvement compared to saline by day 14 indicating an accelerated recovery. By day 28, the behavioral outcome in the saline group was not different compared with both the fresh and frozen MNC groups. Unexpectedly, we did not observe significantly different effects between the two MNC groups at any time points (Figures 2(a) and 2(b)). Overall, both fresh and frozen MNCs improved recovery after stroke but there was no significant difference between two MNCs groups.

### 3.4. Histological Outcomes

**Figure 3:**
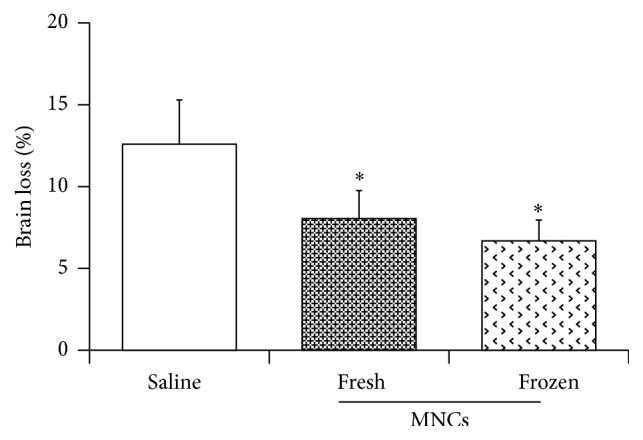
The bar graph exhibiting significantly reduced cerebral atrophy by both fresh MNCs and frozen MNCs at 28 days after stroke. Data are mean ± SD. *n* = 10 in saline group, *n* = 8 in fresh MNCs group, and *n* = 8 in frozen MNCs group. *∗*: *p* < 0.05 compared with saline controls.

Since MNCs have been reported by us and other investigators to exert neuroprotective effects in peri-infarct areas, we measured the lesion size to evaluate any difference caused by cryopreservation. Brain tissue loss estimations of the mice revealed that there was a decrease in atrophy in animals treated with MNCs, irrespective of cryopreservation, compared with saline controls. There was no significant difference in the salvage of brain atrophy when using fresh or frozen MNCs (Figure 3).

## 4. Discussion

Cell based therapies have emerged as a promising candidate in the field of regenerative medicine with recent studies showing promising results in both animals and humans [6, 7, 25, 26]. With an ever-increasing use of cell based therapies, especially those that use autologous cells, there is a need for cryopreservation of these cells for use at a later date. Cryopreservation eliminates the constant need of fresh tissues and enables the use of standardized cell types and preparations over a period of time [12]. However, cryopreservation of cells also brings its own challenges [27]. Effects of cryopreservation on the integrity of cells have been studied by many researchers. In an article published in 1970, Mazur described various factors that can cause injury to cells during cryopreservation [28]. Even though cryopreservation has been studied in detail over the last four decades, there are conflicting results leading to positive and negative consequences [27, 29, 30]. In the present study, we evaluated the effects of cryopreservation on the cellular viability, composition, and phenotype of the various subpopulations of mice bone marrow mononuclear cells (BM-MNCs).

We found that long-term cryopreservation for a year negatively impacted the cellular viability of bone marrow MNCs. Fresh MNCs had a significantly higher viability as compared to cryopreserved MNCs. Our results are consistent with other studies done in different cell types [31, 32]. Cryopreservation also alters the cellular composition of various subpopulations within the MNCs. Specifically, the ratio of T cells within the total number of cells is reduced while NK cells are proportionally increased. Other cell populations such as monocytes were not altered. Considering that certain subpopulations or their ratio may contribute to the reported efficacy of MNCs in various stroke models, the altered composition of MNCs due to cryopreservation may lead to different outcomes in preclinical studies or conceivably in clinical trials. However, despite the changes observed in cryopreserved cells, both fresh and frozen MNCs had similar beneficial effect on behavioral and histological outcomes. These results are also consistent with studies that show that cryopreservation does not affect the functionality of cells [30, 33, 34].

One possibility to consider may be that the lymphocytes within MNCs may not have an important impact on outcome after stroke. The cell populations that were not changed may be more important contributors to the underlying effects of MNCs in rodent models. Specifically, monocytes and progenitor cell populations may be critical cell types within MNCs.

While our results raise the importance of testing the effects of cryopreservation, this study has a number of limitations. Since MNCs in mice are quite different from human bone marrow MNCs, extrapolating to clinical conditions should proceed with caution. In addition, we did not test different cryopreserved condition media.

Overall, our results are consistent with other studies [30, 33] which show that even though viability of the cryopreserved cells is decreased, their treatment effects are not compromised and they are still equally effective as compared to fresh cells. A study by Reich-Slotky et al. showed that cryopreserving CD34+ selected peripheral blood stem cells did not have any adverse outcome on the efficacy of these cells as compared to freshly prepared CD34+ cells [30]. Another study using cryopreserved umbilical cord blood cells showed that they were indeed vasoprotective and showed better responses to cytokines and vascular injury [33]. Many other studies with different cell types have also demonstrated similar results [34, 35]. However, Weise et al. found that cryopreserved human umbilical cord blood mononuclear cells did not show benefits in recovery after experimental stroke in spontaneously hypertensive rats [36]. In that study, the authors did not compare fresh human UCB-MNCs with cryopreserved UCB-MNCs effects and did not provide the difference in characterization between fresh and cryopreserved UCB-MNCs.

## 5. Conclusion

Cryopreservation reduced the viability and altered the composition of BM-MNCs; however, cryopreservation did not change the beneficial effects on recovery after stroke by BM-MNCs in the mouse model. Future investigations should be aimed at increasing the viability of frozen BM-MNCs to optimize their use for regenerative medicine applications [27, 37, 38].

## References

[1] Savitz S. I. (2013). Cell therapies: careful translation from animals to patients. *Stroke*.

[2] Taguchi A., Sakai C., Soma T. (2015). Intravenous autologous bone marrow mononuclear cell transplantation for stroke: phase1/2a clinical trial in a homogeneous group of stroke patients. *Stem Cells and Development*.

[3] Iihoshi S., Honmou O., Houkin K., Hashi K., Kocsis J. D. (2004). A therapeutic window for intravenous administration of autologous bone marrow after cerebral ischemia in adult rats. *Brain Research*.

[4] Kamiya N., Ueda M., Igarashi H. (2008). Intra-arterial transplantation of bone marrow mononuclear cells immediately after reperfusion decreases brain injury after focal ischemia in rats. *Life Sciences*.

[5] Brenneman M., Sharma S., Harting M. (2010). Autologous bone marrow mononuclear cells enhance recovery after acute ischemic stroke in young and middle-aged rats. *Journal of Cerebral Blood Flow and Metabolism*.

[6] Moniche F., Gonzalez A., Gonzalez-Marcos J.-R. (2012). Intra-arterial bone marrow mononuclear cells in ischemic stroke: a pilot clinical trial. *Stroke*.

[7] Friedrich M. A. G., Martins M. P., Araújo M. D. (2012). Intra-arterial infusion of autologous bone marrow mononuclear cells in patients with moderate to severe middle cerebral artery acute ischemic stroke. *Cell Transplantation*.

[8] American Academy of Pediatrics Section on Hematology/Oncology, American Academy of Pediatrics Section on Allergy/Immunology, Lubin B. H., Shearer W. T. (2007). Cord blood banking for potential future transplantation. *Pediatrics*.

[9] Sullivan M. J. (2008). Banking on cord blood stem cells. *Nature Reviews Cancer*.

[10] Zhang Y., Zhu H., Jin H. (2015). Impact of cryopreservation duration of 605 units umbilical cord blood on quality of hematopoietic stem cell and outcome of clinical transplantation. *Zhonghua Xue Ye Xue Za Zhi*.

[11] Hubel A., Spindler R., Curtsinger J. M., Lindgren B., Wiederoder S., McKenna D. H. (2015). Postthaw characterization of umbilical cord blood: markers of storage lesion. *Transfusion*.

[12] Marquez-Curtis L. A., Janowska-Wieczorek A., McGann L. E., Elliott J. A. W. (2015). Mesenchymal stromal cells derived from various tissues: biological, clinical and cryopreservation aspects. *Cryobiology*.

[13] Casado-Díaz A., Santiago-Mora R., Jiménez R. (2008). Cryopreserved human bone marrow mononuclear cells as a source of mesenchymal stromal cells: application in osteoporosis research. *Cytotherapy*.

[14] Xie X.-H., Wang X.-L., He Y.-X. (2012). Promotion of bone repair by implantation of cryopreserved bone marrow-derived mononuclear cells in a rabbit model of steroid-associated osteonecrosis. *Arthritis and Rheumatism*.

[15] Longa E. Z., Weinstein P. R., Carlson S., Cummins R. (1989). Reversible middle cerebral artery occlusion without craniectomy in rats. *Stroke*.

[16] Jiang Y., Vaessen B., Lenvik T., Blackstad M., Reyes M., Verfaillie C. M. (2002). Multipotent progenitor cells can be isolated from postnatal murine bone marrow, muscle, and brain. *Experimental Hematology*.

[17] Yang B., Strong R., Sharma S. (2011). Therapeutic time window and dose response of autologous bone marrow mononuclear cells for ischemic stroke. *Journal of Neuroscience Research*.

[18] Ehlers M., Thiel A., Papewalis C. (2014). Enhanced iodine supplementation alters the immune process in a transgenic mouse model for autoimmune thyroiditis. *Thyroid*.

[19] Lai L., Alaverdi N., Maltais L., Morse H. C. (1998). Mouse cell surface antigens: nomenclature and immunophenotyping. *The Journal of Immunology*.

[20] Mishima Y., Ishihara S., Aziz M. M. (2010). Decreased production of interleukin-10 and transforming growth factor-*β* in Toll-like receptor-activated intestinal B cells in SAMP1/Yit mice. *Immunology*.

[21] Silva J. D., Paredes B. D., Araújo I. M. (2014). Effects of bone marrow-derived mononuclear cells from healthy or acute respiratory distress syndrome donors on recipient lung-injured mice. *Critical Care Medicine*.

[22] Arndt K., Grinenko T., Mende N. (2013). CD133 is a modifier of hematopoietic progenitor frequencies but is dispensable for the maintenance of mouse hematopoietic stem cells. *Proceedings of the National Academy of Sciences of the United States of America*.

[23] Schaar K. L., Brenneman M. M., Savitz S. I. (2010). Functional assessments in the rodent stroke model. *Experimental and Translational Stroke Medicine*.

[24] Yang B., Migliati E., Parsha K. (2013). Intra-arterial delivery is not superior to intravenous delivery of autologous bone marrow mononuclear cells in acute ischemic stroke. *Stroke*.

[25] Lee J. S., Hong J. M., Moon G. J. (2010). A long-term follow-up study of intravenous autologous mesenchymal stem cell transplantation in patients with ischemic stroke. *STEM CELLS*.

[26] Bang O. Y., Lee J. S., Lee P. H., Lee G. (2005). Autologous mesenchymal stem cell transplantation in stroke patients. *Annals of Neurology*.

[27] Shu Z., Gao D., Pu L. L. Q. (2015). Update on cryopreservation of adipose tissue and adipose-derived stem cells. *Clinics in Plastic Surgery*.

[28] Mazur P. (1970). Cryobiology: the freezing of biological systems. *Science*.

[29] Medd P., Nagra S., Hollyman D., Craddock C., Malladi R. (2013). Cryopreservation of allogeneic PBSC from related and unrelated donors is associated with delayed platelet engraftment but has no impact on survival. *Bone Marrow Transplantation*.

[30] Reich-Slotky R., Bachegowda L. S., Ancharski M., Gergis U., van Besien K., Cushing M. M. (2015). Engraftment for CD34 selected stem cell products is not compromised by cryopreservation. *Transfusion*.

[31] Rubinstein P., Dobrila L., Rosenfield R. E. (1995). Processing and cryopreservation of placental/umbilical cord blood for unrelated bone marrow reconstitution. *Proceedings of the National Academy of Sciences of the United States of America*.

[32] Silani V., Pizzuti A., Strada O., Falini A., Buscaglia M., Scarlato G. (1988). Human neuronal cell viability demonstrated in culture after cryopreservation. *Brain Research*.

[33] Yin Y., Liu H., Wang F. (2015). Transplantation of cryopreserved human umbilical cord blood-derived endothelial progenitor cells induces recovery of carotid artery injury in nude rats. *Stem Cell Research and Therapy*.

[34] Kanatsu-Shinohara M., Ogonuki N., Inoue K., Ogura A., Toyokuni S., Shinohara T. (2003). Restoration of fertility in infertile mice by transplantation of cryopreserved male germline stem cells. *Human Reproduction*.

[35] Aird W., Labopin M., Gorin N. C., Antin J. H. (1992). Long-term cryopreservation of human stem cells. *Bone Marrow Transplantation*.

[36] Weise G., Lorenz M., Pösel C. (2014). Transplantation of cryopreserved human umbilical cord blood mononuclear cells does not induce sustained recovery after experimental stroke in spontaneously hypertensive rats. *Journal of Cerebral Blood Flow and Metabolism*.

[37] Chen G., Yue A., Ruan Z. (2016). Comparison of the effects of different cryoprotectants on stem cells from umbilical cord blood. *Stem Cells International*.

[38] Asghar W., El Assal R., Shafiee H., Anchan R. M., Demirci U. (2014). Preserving human cells for regenerative, reproductive, and transfusion medicine. *Biotechnology Journal*.

